# Performance evaluation of iterative reconstruction algorithms for achieving CT radiation dose reduction — a phantom study

**DOI:** 10.1120/jacmp.v17i2.5709

**Published:** 2016-03-08

**Authors:** Cristina T. Dodge, Eric P. Tamm, Dianna D. Cody, Xinming Liu, Corey T. Jensen, Wei Wei, Vikas Kundra, X. John Rong

**Affiliations:** ^1^ Department of Imaging Physics The University of Texas MD Anderson Cancer Center Houston TX USA; ^2^ Department of Diagnostic Radiology The University of Texas MD Anderson Cancer Center Houston TX USA; ^3^ Department of Biostatistics The University of Texas MD Anderson Cancer Center Houston TX USA; ^4^ Department of Cancer Systems Imaging The University of Texas MD Anderson Cancer Center Houston TX USA

**Keywords:** CT, iterative reconstruction, image quality, radiation dose

## Abstract

The purpose of this study was to characterize image quality and dose performance with GE CT iterative reconstruction techniques, adaptive statistical iterative reconstruction (ASiR), and model‐based iterative reconstruction (MBIR), over a range of typical to low‐dose intervals using the Catphan 600 and the anthropomorphic Kyoto Kagaku abdomen phantoms. The scope of the project was to quantitatively describe the advantages and limitations of these approaches. The Catphan 600 phantom, supplemented with a fat‐equivalent oval ring, was scanned using a GE Discovery HD750 scanner at 120 kVp, 0.8 s rotation time, and pitch factors of 0.516, 0.984, and 1.375. The mA was selected for each pitch factor to achieve ${\text{CTDI}}_{\text{vol}}$ values of 24, 18, 12, 6, 3, 2, and 1 mGy. Images were reconstructed at 2.5 mm thickness with filtered back‐projection (FBP); 20%, 40%, and 70% ASiR; and MBIR. The potential for dose reduction and low‐contrast detectability were evaluated from noise and contrast‐to‐noise ratio (CNR) measurements in the CTP 404 module of the Catphan. Hounsfield units (HUs) of several materials were evaluated from the cylinder inserts in the CTP 404 module, and the modulation transfer function (MTF) was calculated from the air insert. The results were confirmed in the anthropomorphic Kyoto Kagaku abdomen phantom at 6, 3, 2, and 1 mGy. MBIR reduced noise levels five‐fold and increased CNR by a factor of five compared to FBP below 6 mGy ${\text{CTDI}}_{\text{vol}}$, resulting in a substantial improvement in image quality. Compared to ASiR and FBP, HU in images reconstructed with MBIR were consistently lower, and this discrepancy was reversed by higher pitch factors in some materials. MBIR improved the conspicuity of the high‐contrast spatial resolution bar pattern, and MTF quantification confirmed the superior spatial resolution performance of MBIR versus FBP and ASiR at higher dose levels. While ASiR and FBP were relatively insensitive to changes in dose and pitch, the spatial resolution for MBIR improved with increasing dose and pitch. Unlike FBP, MBIR and ASiR may have the potential for patient imaging at around 1 mGy ${\text{CTDI}}_{\text{vol}}$. The improved low‐contrast detectability observed with MBIR, especially at low‐dose levels, indicate the potential for considerable dose reduction.

PACS number(s): 87.57.Q‐, 87.57,nf, 87.57.C‐, 87.57.cj, 87.57.cf, 87.57.cm, 87.57.uq

## I. INTRODUCTION

With the introduction of computed tomography (CT) in the 1970s, complex analytical approaches were conceived to reconstruct the scanned image. Algebraic Reconstruction Technique (ART) was the first image reconstruction method proposed for CT.^1^, ^2^, ^3^ Computational limitations and large volumes of raw data led to the development of alternative reconstruction techniques, such as filtered back‐projection (FBP). The simplified assumptions (pencil beam, point source, point detector) of FBP do not account for quantum and electronic noise^(4)^ in the raw data. Furthermore, noise is further amplified by the filter used in the reconstruction process.^(1)^

The latest developments in CT reconstruction techniques, adaptive statistical iterative reconstruction (ASiR, GE Healthcare, Waukesha, WI) and model‐based iterative reconstruction (MBIR, GE Healthcare, Waukesha, WI),^(5)^ can sort through the raw data and preferentially weight projections containing statistically reliable information to reduce noise. Modern computational techniques make these iterative reconstruction techniques clinically feasible and allow the implementation low‐dose protocols. ASiR employs 2D edge‐preserving, noise‐reducing algorithms to filter projection data. Assuming Poisson statistics of noise, the technique filters out highly attenuated projections based on uncertainties in their statistics, resulting in a loss of information and spatial resolution, which are somewhat mitigated by the ability to blend the results with FBP.^3^, ^6^ MBIR extends ASiR methods by anticipating the precursors of image noise. This is accomplished by modeling the properties of noise, accounting for photon flux and for noise introduced by scanner electronics (photodiode and the digitization process).^(7)^ The model also extends to system geometry and statistics; MBIR takes into consideration the X‐ray energy spectrum, beam hardening, and divergence, which are determined using Monte Carlo models to precompute the interactions of a polychromatic X‐ray beam with the detector system and various tissues. Further geometrical modeling estimates the amount of image degradation caused by the finite size of the focal spot and detector elements.^7^, ^8^ The accuracy of these models has great bearing on the resulting images.^(1)^

In this study, we have evaluated the performance of both ASiR and MBIR in a clinical setting for achieving potential patient dose reduction. We scanned the Catphan 600 modular phantom, supplemented with a fat‐equivalent oval ring, using standard clinical scan parameters at our institution for a medium adult, modified to evaluate image noise, contrast, material Hounsfield unit (HU), and resolution across seven dose levels and three pitch factors. To account for the dependence of tube‐current modulation and iterative reconstruction on the shape and size of the patient, the results were confirmed with a Kyoto Kagaku anthropomorphic abdomen phantom. The evaluation of FBP, ASiR, and MBIR was performed under controlled, reproducible and clinically relevant scan conditions.

## II. MATERIALS AND METHODS

### A. Phantom scan and reconstruction parameters

**Figure 1:**
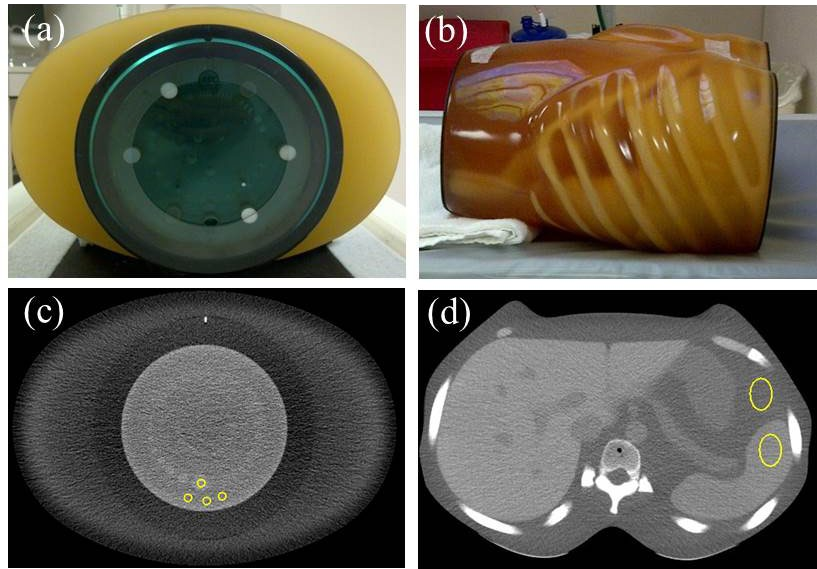
The Catphan 600 phantom surrounded by the fat‐equivalent ring (a) and the Kyoto Kagaku abdomen phantom (b). The locations of the ROIs used for signal and noise measurements are indicated in the Catphan (c) and abdomen phantom (d) images.

**Figure 2:**
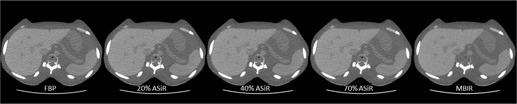
For illustration purpose, images of the Kyoto Kagaku phantom acquired at 3 mGy ${\text{CTDI}}_{\text{vol}}$ and reconstructed with the three different algorithms: FBP, ASiR, and MBIR.

Catphan 600 phantom (The Phantom Laboratory, Inc., Salem, NY) and Kyoto Kagaku abdomen phantom (Kyoto Kagaku Co., Ltd., Kyoto, Japan) images were acquired on a 64‐detector row CT scanner (Discovery CT750HD, GE Healthcare): 120 kVp, 0.8 s rotation time, 40 mm beam width, large scan field‐of‐view (SFOV), 2.5 mm image thickness, and three pitch factors (0.516, 0.984, and 1.375). For the Catphan phantom, the mAs was selected for each pitch factor to achieve 1, 2, 3, 6, 12, 18, and 24 mGy ${\text{CTDI}}_{\text{vol}}$ values. During the scans, each phantom module (CTP515 for low‐contrast detectability, CTP528 for high‐contrast, and CTP404 for material HU) was supplemented with a fat‐equivalent oval ring to better approximate an adult body shape and size, as shown in Fig. 1(a). For the Kyoto Kagaku abdomen phantom, the mAs was varied to achieve reported ${\text{CTDI}}_{\text{vol}}$ of 1, 2, 3, and 6 mGy. Images were reconstructed using the Standard kernel for FBP; 20%, 40%, and 70% ASiR, which represent a wide range of percentage blends of the ASiR with the FBP; and MBIR. Figure 2 illustrates images of the Kyoto Kagaku abdomen phantom acquired at 3 mGy ${\text{CTDI}}_{\text{vol}}$ and reconstructed using different algorithms. The display field‐of‐view (DFOV) was 36 cm for the reconstructed images of Catphan phantom and was 30 cm for the reconstructed images of Kyoto Kagaku abdomen phantom. These default DFOV sizes were selected based on the lateral dimensions of the phantoms from skin‐to‐skin. The image matrix was 512×512.

### B. Image quality metrics

To ensure that the data is sampled from images approximating clinical conditions, all quantitative image analysis was performed directly from the images with default DFOVs, without reconstructing to a small DFOV. To minimize statistical variations in noise for each scan condition, we analyzed images from 10 independent acquisitions at the same identical location in a phantom. ImageJ software (U.S. National Institutes of Health, Bethesda, Maryland)^(9)^ was used to analyze the phantom images downloaded from PACS (iSite Enterprise, Philips Healthcare, Andover, MA).

#### B.1 Low‐contrast detectability: noise

Noise was calculated as the mean of the standard deviations of three, $0.4\;{\text{cm}}^{2}$ regions of interest (ROIs) located in the background material of the Catphan. Three ROIs increased the total area of data sampling (from 0.4 to $1.2\;{\text{cm}}^{2}$). For the Kyoto Kagaku abdomen phantom image analysis, one $3.1\;{\text{cm}}^{2}$ ROI was used (Fig. 1).

#### B.2 Low‐contrast detectability: contrast‐to‐noise ratio

To calculate the contrast‐to‐noise ratio (CNR), an ROI was placed in the center of the 1% contrast, 15 mm diameter supraslice target of the Catphan CTP515 module and three identical ROIs were placed in the immediate background. The CNR was defined as the mean target signal minus the mean background signal divided by the background standard deviation. For the Kyoto Kagaku abdomen phantom, the target organs used were the spleen, liver, pancreas, and kidneys. Figures 1(c) and (d) show that, in both cases, the sizes of the ROIs in the target and background were identical.

(1) $$CNR=\frac{\left|Object\;HU-Background\;HU\right|}{Background\;\text{Standard}\;Deviation}$$

#### B.3 Changes in image quality with dose and reconstruction techniques

The difference in noise and CNR between the three techniques (namely FBP, ASiR, and MBIR) was calculated. To evaluate changes in image quality with dose, a baseline reference value was defined as the noise or CNR achieved with MBIR at 1 mGy. The 1 mGy ${\text{CTDI}}_{\text{vol}}$ value was chosen to represent a very low‐dose target level and is consistent with several recent clinical CT imaging studies where ${\text{CTDI}}_{\text{vol}}$ levels near or at 1 mGy were evaluated.^10^, ^11^, ^12^

#### B.4 High‐contrast spatial resolution

**Figure 3:**
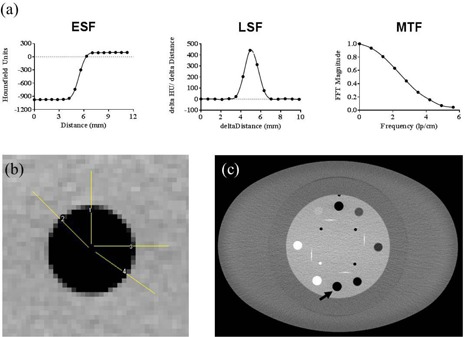
Stylized ESF, LSF and MTF graphs (a) depict the steps in the MTF calculation process. The orientation of the four line profiles (b) traversing the air target of the CTP404 module surrounded the fat‐equivalent ring. The black arrow in (c) points to the location of the air insert used for MTF analysis.

Visual inspection of the CTP528 high resolution module of the Catphan 600 was used to evaluate gross changes in spatial resolution, as typically performed during annual compliance testing. The air target in the CTP404 module was used to measure the modulation transfer function (MTF) of reconstructed images using the edge method as depicted in Fig. 3.^13^, ^14^, ^15^ Fourteen mm line profiles that started at the center of the target and traversed an equal amount of surrounding material were used to obtain the edge‐spread function (ESF). Two of the profiles sample the pixels at the edge of the target horizontally and two diagonally, to avoid bias in the sampling. The line‐spread function (LSF) was calculated as the derivative of the ESF. The magnitude of the fast Fourier transform (FFT) of the LSF was normalized to zero frequency to calculate the MTF. FFT magnitude results from 10 replicate images obtained at the same location in the phantom and four line profiles were added together to smooth the MTF.

#### B.5 Hounsfield unit change

Eight cylindrical inserts in the CTP404 module of the Catphan were used to quantify changes in HU: air, PMP, LDPE, water, polystyrene, acrylic, Delrin, and Teflon. Slight variations from the estimated CT numbers in phantom manual were expected to result from use of the fat ring during scan acquisition.

### C. Statistical analysis

Noise, CNR, and MTF were summarized using mean, standard deviation (SD), and range. Noise and MTF were transformed to the logarithmic scale prior to statistical modeling. For noise, a positive difference means higher noise (worse) and a negative means lower noise (better). For CNR, a positive difference means higher CNR (better), while a negative difference means lower CNR (worse). Logarithmic transformation is to reduce skewness in the data to better suit the underlying Normal assumption of linear mixed model or ANOVA. Linear mixed model was used to estimate and compare noise and CNR between algorithms. Linear mixed model can account for correlations between measurements from the same experimental unit. As ANOVA is a generalized form of two‐sample *t*‐test, the linear mixed model is a generalized form of paired *t*‐test. The interpretation of linear mixed model is the same as ANOVA. Dunnett's procedure was used to adjust for multiple pairwise comparisons against the reference level (MBIR at ${\text{CTDI}}_{\text{vol}}$ of 1 mGy). ANOVA was used to compare MTF between algorithms by pitch, ${\text{CTDI}}_{\text{vol}}$, and spatial frequency. Pairwise comparisons against FBP, based on ANOVA estimates, were also adjusted using the Dunnett's procedure. All tests were two‐sided and adjusted p‐values of 0.05 or less were considered statistically significant. Statistical analysis was carried out using SAS version 9 (SAS Institute, Cary, NC). The values of statistical analysis (e.g., adjusted p‐value) have been associated to the outcomes in Appendix A.

## III. RESULTS

### A. Low‐contrast detectability: noise and CNR

**Figure 4:**
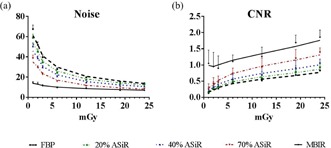
Noise (a) and CNR (b) plotted as a function of increasing dose for FBP (black dashed line), ASiR (colored dashed lines), and MBIR (solid line) at pitch 0.984. Analysis performed on images acquired with the Catphan 600 phantom.

**Figure 5:**
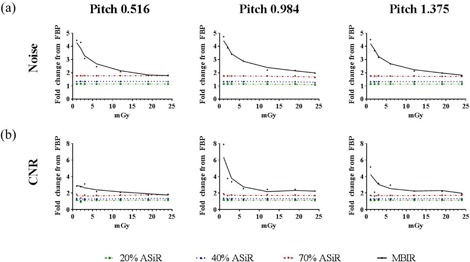
Fold improvement in (a) noise and (b) CNR plotted as a function of increasing dose and pitch factor for ASiR (dashed lines) and MBIR (solid line) relative to FBP. Analysis performed on images acquired with the Catphan 600 phantom.

**Figure 6:**
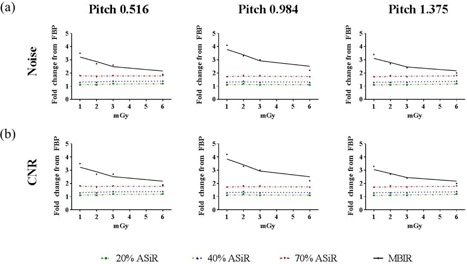
Fold improvement in (a) noise and (b) CNR plotted as a function of increasing dose and pitch factor for ASiR (dashed lines) and MBIR (solid line) relative to FBP. Representative data from the analysis performed on the liver region of the Kyoto Kagaku abdomen phantom.

Noise and CNR analysis with the Catphan 600 clearly demonstrated the advantage of using MBIR, especially at low‐dose levels (Fig. 4). Compared to the moderate improvement in CNR observed with ASiR (1.2 times for 20% ASiR, 1.3 times for 40% ASiR, and 1.8 times for 70% ASiR), MBIR resulted in a five times increase in CNR at low‐dose levels, relative to FBP. Above 5 mGy ${\text{CTDI}}_{\text{vol}}$, the improvements observed with MBIR were within experimental error to those of 70% ASiR. Noise reduction with MBIR was constant among the three pitch factors and similar in magnitude to the improvements observed with CNR (Fig. 5(a)). However, the CNR did vary with pitch when MBIR was used: eightfold for 0.984, fivefold for 1.375, and threefold for 0.516 (Fig. 5(b)). Likewise, with the Kyoto Kagaku abdomen phantom, a mean three times reduction in noise and three times improvement in CNR were achieved for the four organs investigated (Fig. 6). A similar trend of slightly increased CNR at a 0.984 pitch was observed with the abdomen phantom.

### B. Changes in noise and CNR with dose and reconstruction techniques

**Figure 7:**
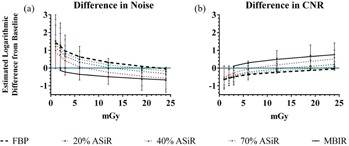
Summary of estimated noise and CNR difference between reconstruction technique/${\text{CTDI}}_{\text{vol}}$ combinations and the baseline, reference value (MBIR at ${\text{CTDI}}_{\text{vol}}$ of 1 mGy), plotted as the mean ±95% CI. (a) For noise, a positive difference (above the blue line) means higher noise (worse) and a negative means lower noise (better). (b) For CNR, a positive difference (above the blue line) means higher CNR (better), a negative difference means lower CNR (worse).

Estimated noise and CNR differences from a baseline, reference level were calculated to quantify changes in image quality with increasing ${\text{CTDI}}_{\text{vol}}$ across the reconstruction techniques (Fig. 7). From the estimated noise differences (Fig. 7(a)), it can be observed that 40% ASiR and 70% ASiR at 12 mGy ${\text{CTDI}}_{\text{vol}}$ and 20% ASiR and FBP at 18 mGy ${\text{CTDI}}_{\text{vol}}$ yielded a similar noise as MBIR at 1 mGy ${\text{CTDI}}_{\text{vol}}$. On the other hand, from the estimated CNR differences (Fig. 7(b)), the following results are evident: a) MBIR yielded similar CNR at 1, 2, and 3 mGy ${\text{CTDI}}_{\text{vol}}$; b) 70% ASiR reached the baseline CNR at ${\text{CTDI}}_{\text{vol}}$ of 6 mGy and it yielded significantly better CNR at higher doses (adjusted p‐value=0.70); c) 20% ASiR and 40% ASiR CNR values at 12 mGy were comparable to MBIR CNR at 1 mGy and didn't yield significantly better CNR until ${\text{CTDI}}_{\text{vol}}$ reached 24 mGy (adjusted p‐value=0.48 and 1.00, respectively); d) FBP needed to reach 18 mGy ${\text{CTDI}}_{\text{vol}}$ to yield similar CNR as MBIR at 1 mGy ${\text{CTDI}}_{\text{vol}}$, and it didn't yield significantly better CNR even at ${\text{CTDI}}_{\text{vol}}$ of 24 mGy.

### C. High‐contrast spatial resolution

**Figure 8:**
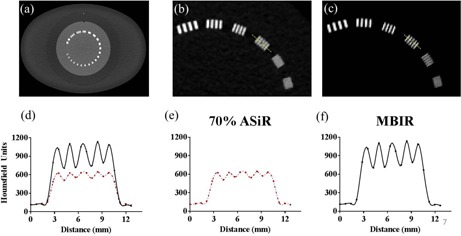
Spatial resolution evaluated from the CTP528 high‐resolution module (a) of the Catphan 600 phantom, scanned with 11 mGy and 1.375 pitch. Overlapping (d) or single profiles for 70% ASiR (e) and MBIR (f) from lines drawn across the 7 lp/mm bar pattern, as depicted in (b) and (c), respectively. For the same scan parameters, the spatial resolution of MBIR is superior as observed from the bar pattern analysis.

**Figure 9:**
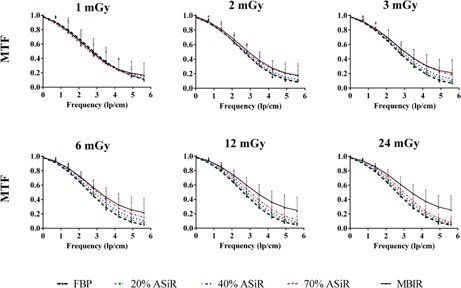
The MTF plotted as a function of increasing spatial frequency for FBP (black dashed lines), ASiR (colored dashed lines), and MBIR (solid line) at 0.984 pitch and 1, 2, 3, 6, 12 and 24 mGy. Analysis performed on images acquired with the Catphan 600 phantom.

**Figure 10:**
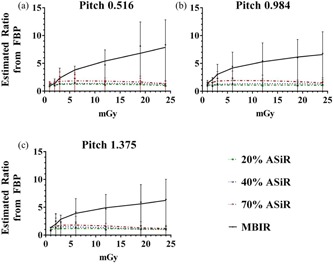
Summary of estimated ratios between ASiR/MBIR and FBP with respect to MTF by pitch and ${\text{CTDI}}_{\text{vol}}$ at 5 lp/cm, plotted as mean ±95% CI. MTF was transformed to the logarithmic scale prior to ANOVA analysis. Estimated differences on the logarithmic scale were back‐transformed as ratio to the raw scale as shown in the table. Dunnett's adjustment was used to control overall type 1 error rate at 5% for each model. Detailed results of the statistical analysis can be found in Appendix A.

To evaluate spatial resolution, an initial inspection of bar patterns demonstrated an improvement with iterative reconstruction techniques; this observation was supported by line profiles drawn across the 7 lp/cm bar pattern (Fig. 8). The MTF was also computed for the various reconstruction algorithms and dose levels. Figure 9 shows the improved MTF of MBIR at pitch 0.984, with increasing dose and frequency compared to ASiR and FBP. To compare the MTF for MBIR and ASiR to FBP at a spatial frequency of 5 lp/cm, the estimated ratio relative to FBP was calculated and the results are plotted in Fig. 10. Estimated mean ratio higher than 1 means larger MTF than FBP, and lower than 1 means smaller MTF than FBP. For example, MTF of 20% ASiR at setting of pitch 0.516, dose 0.89 mGy was 0.86 of FBP (95% CI: 0.45–1.63). At pitch factors of 0.516 and 0.984, MBIR yielded significantly higher MTF than FBP above 3 mGy ${\text{CTDI}}_{\text{vol}}$. At pitch 1.375, the dose threshold for significant improvement in MTF with MBIR was 2 mGy. Seventy percent (70%) ASiR showed significantly higher MTF than FBP only at 3 mGy for pitch 0.516, 3 and 6 mGy for pitch 0.984, and at 2 and 6 mGy for pitch 1.375. Twenty percent (20%) ASiR and 40% ASiR MTF were not significantly different from FBP MTF at any dose level for all pitch factors.

### D. Hounsfield unit change

**Figure 11:**
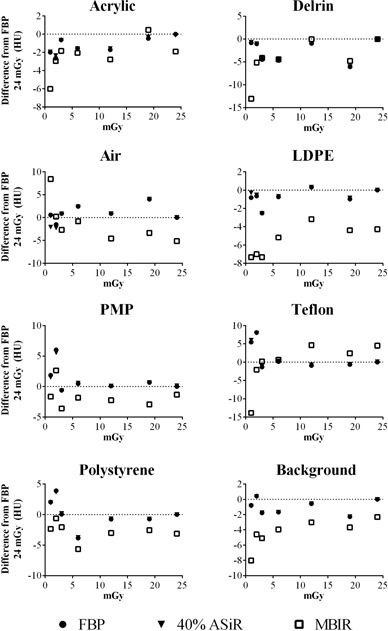
The difference in HU from FBP at 24 mGy, for all eight materials in the Catphan sensitometry module, is plotted as a function of increasing dose for FBP (circles), ASiR (triangles), and MBIR (squares) at 0.984 pitch. For clarity and given the similarity in results for the ASiR blends, 40% ASiR was chosen as representative data.

Given their impact on clinical diagnosis, the effects of iterative reconstruction on HU were evaluated. For all eight materials in the sensitometry module of the Catphan 600 phantom — background material (100 HU), PMP (−200 HU), LDPE (−100 HU), polystyrene (−35 HU), acrylic (120 HU), Delrin (340 HU), Teflon (990 HU), and air (−1000 HU) — the material HU values in MBIR images were closer overall to those listed in the Catphan 600 user manual than the HU values in ASiR and FBP images. Table A6 in Appendix A summarizes changes in material HUs with reconstruction algorithm and dose levels. As deviations from the HUs estimated in the Catphan 600 user manual are expected from the use of the fat‐equivalent ring, the data were also plotted as the difference in HU from images reconstructed with FBP at 24 mGy (Fig. 11). The material HU values in MBIR images are on average 10 HU below those of FBP, whereas the material HU values in 40% ASiR images are almost identical to those in the FBP images. Figure 11 also shows that below 5 mGy ${\text{CTDI}}_{\text{vol}}$, the measured material HU values differed greatly from the HU values of the FBP images at 24 mGy ${\text{CTDI}}_{\text{vol}}$ level.

## IV. DISCUSSION

Since adaptive statistical iterative reconstruction has become available for clinical patient imaging, the potential for dose reduction with ASiR has been extensively studied in the literature. The reported dose reduction ranges from 30%–60%. In pediatric imaging (aged 1‐year‐old to adolescence), 100% ASiR was estimated to reduce dose by 82% compared to FBP in a phantom study.^(6)^ Later on, 40% ASiR was implemented clinically, with 42%–48% dose reductions observed.^(6)^ In CT ACR phantom studies,^(6)^ a dose reduction potential of 25%–29% has been reported, when the vendor‐recommended 30% ASiR was applied. In chest exams of an elderly patient population (60 years±15), Leipsic *et al*.^(16)^ reported a 26% dose reduction when 30% ASiR was applied.

The modest reduction in dose previously reported confirms that ASiR was not intended to result in marked dose reduction, but was rather a balanced approach to maintain image quality with reasonable dose savings.^(17)^ The improvement in image quality observed when correctly modeling the noise properties of the image has led to the development of model‐based iterative reconstruction techniques that model the X‐ray and image production chain. Several publications have reported that MBIR resulted in dose reductions of 50%–75% in abdominal CT,^(18)^ 70%–80% in chest CT,^19^, ^20^ and 67%–86% in phantom studies.^(21)^ For the same delivered dose, MBIR increases the SNR and CNR compared to FBP, a 30% decrease in noise and 46% increase in CNR on abdominal imaging^(4)^ and 70% and 60% decreases in noise in paranasal CT^(22)^ and phantom studies, respectively.^(23)^ In agreement with our findings, Husarik *et al*.^(18)^ reported that, in abdominal liver examinations with ${\text{CTDI}}_{\text{vol}}$ of 4.38−23.35 mGy, an increase in CNR (1.5–2.7 CNR for MBIR and 0.16–0.63 CNR for FBP, medium patient, 120 kVp) and an 80% decrease in noise with MBIR compared with FBP were observed. Shuman *et al*.^(24)^ also reported in a liver study that image background noise with MBIR was significantly lower and CNR was significantly higher compared to FBP and ASiR of the same raw dataset, and hence at the same dose level of clinical liver imaging. Furthermore, in the cervicothoracic region, which suffers from noise and streak artifacts as a result of beam hardening through the shoulders, Katsura et al.^(25)^ reported that MBIR improved both noise and spatial resolution, whereas the high‐ and low‐pass filters used in analytical reconstruction techniques only recovered one or the other.

A phantom study provides the opportunity for performing in‐depth evaluations, by adjusting one scan/reconstruction parameter at a time, while other conditions remain unchanged, so that appropriate comparisons are conducted. Such work is especially valuable when a phantom is scanned repeatedly at many different dose levels for assessing the potential for dose reduction. In contrast with other phantom studies, the phantoms used in our work mimicked patient size/ shape, the base scan protocol was a routine clinical protocol used for patient abdominal imaging, and the phantom images were reconstructed with clinical parameters. We therefore performed an analysis of image quality under various conditions of image reconstruction and at a number of radiation dose levels. The quantitative image analysis included noise, CNR, material HU, and spatial resolution. Our results show that the largest improvement in noise reduction and contrast with MBIR (threefold to fivefold) occurred at the lowest dose levels, demonstrating the potential feasibility of low‐dose patient imaging where the appropriate dose levels depend on clinical applications as well as sizes of patients. Conversely, noise, CNR, and material HU for three ASiR blends are less dependent on dose level and provide for a more modest reduction in dose, 15%–20%.

However, image analysis based on noise and CNR does not completely capture the differences in image texture, which may affect the outcomes of patient diagnosis. This limitation of the study could account for the differences in results presented here compared to published clinical studies. Additionally, the use of clinical scan parameters, the size/shape and attenuation of the phantoms (added a fat ring to the Catphan 600 for approximating a medium size patient and the anthropomorphic abdomen phantom), the data sampling directly from the large DFOV, plus additional validation with an anthropomorphic phantom might also account for the discrepancy in results. Overall, this study is an initial step in the systematic evaluation of iterative reconstructions and is limited by the use of phantom data and objective ROIs. Depending on clinical applications, future task‐based image quality assessment will be conducted to evaluate overall image quality including, but not limited to, texture characteristics and spatial resolution at various contrast levels.

Regarding if/how iterative reconstructions affect material HU, there exist a small number of publications. Using the Catphan 600 phantom at 1 mGy ${\text{CTDI}}_{\text{vol}}$, 120 kVp, and 1.375 pitch, Mieville *et al*.^(21)^ reported no HU differences for the air and PMP inserts, differences of 3–4 HU for polystyrene and Delrin, and differences of 10 HU for Teflon for MBIR compared to 100% ASiR and FBP. Larger differences have been reported between MBIR and FBP — 19–20 HU for Delrin and 31–33 HU for Teflon — using the Catphan 600 and a bone‐mimicking ring.^(26)^ In our study, all three ASiR blends behaved similar to FBP, whereas MBIR reconstructed images had HU that were on average 10 HU lower than the FBP HU at 24 mGy. All these results indicate that MBIR affects HU, especially for materials above 200 HU (Teflon and Delrin).

It must be emphasized that the results presented here must be considered alongside the limitations of the methodology used to evaluate the reconstruction approaches. Although contrast and noise are basic properties of image quality, there are other metrics, such as the Fourier‐based noise power spectrum (NPS), that may provide characterization of additional dimensions of image quality by taking into account the multifaceted properties of noise and image texture. However, it can also be argued that the application of FFT to FBP and iterative reconstructed images, such as for the purposes of MTF or NPS analysis, is not appropriate because the basic assumptions of linearity and shift‐invariance are violated, especially for MBIR images. In the case of FBP, the assumptions come reasonably close and the scientific community has adopted the use of FFT methods.^(27)^ Conversely, given the spatial‐dependence of noise and the contrast‐dependence of spatial resolution introduced by iterative reconstruction, it is difficult to argue for the shift‐invariance of images reconstructed with iterative methods.^(28)^

Therefore, a perceptional reader study is perhaps a more appropriate option for making a direct comparison of images reconstructed using FBP and iterative reconstruction methods. There are reports that, even though the apparent difference in the texture of the MBIR images makes it difficult to conduct a blinded reader study,^(19)^ the appearance of MBIR images is reported to have a minimal impact on clinical diagnosis.^19^, ^29^, ^30^ In the near future, we plan to conduct a reader study based on the phantom images that we have acquired to evaluate the effect of texture on perception, but it is not within the scope of this study. It must be emphasized that the results presented here apply to the very specific phantom/image acquisition conditions and objects analyzed, and the results may not be readily extrapolated to other situations.

## V. CONCLUSIONS

We performed an objective comparison of FBP, ASiR, and MBIR using both modular and anthropomorphic phantoms over a wide range of image acquisition conditions: 3 levels of ASiR, 3 pitch factors, and 6–7 dose levels. Our results show that iterative reconstruction produced low‐dose images that are equivalent in image quality to that of conventional FBP images acquired at higher dose levels. In addition, HUs in MBIR images are highly sensitive to low‐dose levels, requiring careful attention for quantitative assessment of anatomy. A combined effort by clinical staff, radiologists, and medical physicists will be needed to integrate these findings into the clinical workflow and establish new CT protocols and standard operating procedures.

## ACKNOWLEDGMENTS

This work was performed in collaboration with General Electric Healthcare Technologies at the Center for Advanced Biomedical Imaging, University of Texas MD Anderson Cancer Center.

## COPYRIGHT

This work is licensed under a Creative Commons Attribution 4.0 International License.

## APPENDICES

### Appendix A: Statistical Model Estimates

**Table 1:** Table A1. Summary of estimated mean noise by Recon and ${\text{CTDI}}_{\text{vol}}$. Noise was transformed to the logarithmic scale before being analyzed by linear mixed model. For example, VEO at ${\text{CTDI}}_{\text{vol}}$ 1 yielded a mean noise of 2.68 (95% CI: 2.65–2.70), whereas FBP at ${\text{CTDI}}_{\text{vol}}$ of 1 yielded a mean noise of 4.20 (95% CI: 4.17–4.22)

*Recon*	${\text{CTDI}}_{\text{vol}}$	*Estimated Mean Noise (logarithmic scale)*	*95% LCL*	*95% UCL*
20% ASiR	1	4.06	4.03	4.09
40% ASiR	1	3.91	3.88	3.93
70% ASiR	1	3.64	3.61	3.66
FBP	1	4.20	4.17	4.22
VEO	1	2.68	2.65	2.70
20% ASiR	1.5	3.82	3.80	3.85
40% ASiR	1.5	3.67	3.64	3.70
70% ASiR	1.5	3.40	3.38	3.43
FBP	1.5	3.96	3.94	3.99
VEO	1.5	2.58	2.56	2.61
20% ASiR	3	3.49	3.46	3.52
40% ASiR	3	3.34	3.31	3.36
70% ASiR	3	3.07	3.04	3.09
FBP	3	3.63	3.60	3.66
VEO	3	2.46	2.43	2.48
20% ASiR	6	3.17	3.14	3.19
40% ASiR	6	3.01	2.98	3.04
70% ASiR	6	2.74	2.72	2.77
FBP	6	3.30	3.28	3.33
VEO	6	2.32	2.29	2.34
20% ASiR	12	2.83	2.80	2.85
40% ASiR	12	2.67	2.64	2.70
70% ASiR	12	2.40	2.37	2.43
FBP	12	2.97	2.94	2.99
VEO	12	2.20	2.18	2.23
20% ASiR	18	2.58	2.55	2.61
40% ASiR	18	2.43	2.40	2.45
70% ASiR	18	2.16	2.13	2.18
FBP	18	2.72	2.69	2.74
VEO	18	2.03	2.00	2.06
20% ASiR	24	2.48	2.45	2.51
40% ASiR	24	2.33	2.30	2.35
70% ASiR	24	2.06	2.04	2.09
FBP	24	2.60	2.57	2.62
VEO	24	1.97	1.95	2.00

**Table 2:** Table A2. Summary of estimated noise difference between Recon/CTDIvol combinations and the reference (VEO at CTDIvol of 1). Estimates were based on linear mixed model and Dunnett's adjustment was used to control overall type I error rate at 5%. A positive difference means higher noise (worse) and a negative means lower noise (better). For example, 20% ASiR at CTDIvol of 1 yielded significantly higher noise than VEO at CTDIvol 1 (difference=1.38, 95% CI: 1.35−1.42, adjusted p‐value<0.0001)

*Recon*	${\text{CTDI}}_{\text{vol}}$	*Estimated Mean Noise Difference (logarithmic scale)*	*95% LCL*	*95% UCL*	*Adjusted p‐value*
20% ASiR	1	1.38	1.35	1.42	<.0001
40% ASiR	1	1.23	1.19	1.27	<.0001
70% ASiR	1	0.96	0.92	1.00	<.0001
FBP	1	1.52	1.48	1.56	<.0001
20% ASiR	1.5	1.15	1.11	1.18	<.0001
40% ASiR	1.5	0.99	0.96	1.03	<.0001
70% ASiR	1.5	0.73	0.69	0.76	<.0001
FBP	1.5	1.29	1.25	1.32	<.0001
VEO	1.5	−0.09	−0.13	−0.06	<.0001
20% ASiR	3	0.81	0.78	0.85	<.0001
40% ASiR	3	0.66	0.62	0.70	<.0001
70% ASiR	3	0.39	0.35	0.43	<.0001
FBP	3	0.95	0.91	0.99	<.0001
VEO	3	−0.22	−0.26	−0.18	<.0001
20% ASiR	6	0.49	0.45	0.53	<.0001
40% ASiR	6	0.33	0.30	0.37	<.0001
70% ASiR	6	0.06	0.03	0.10	0.02
FBP	6	0.63	0.59	0.66	<.0001
VEO	6	−0.36	−0.40	−0.32	<.0001
20% ASiR	12	0.15	0.11	0.19	<.0001
40% ASiR	12	−0.01	−0.04	0.03	1.00
70% ASiR	12	−0.28	−0.31	−0.24	<.0001
FBP	12	0.29	0.25	0.32	<.0001
VEO	12	−0.48	−0.51	−0.44	<.0001
20% ASiR	18	−0.10	−0.13	−0.06	<.0001
40% ASiR	18	−0.25	−0.29	−0.22	<.0001
70% ASiR	18	−0.52	−0.56	−0.48	<.0001
FBP	18	0.04	0.003	0.08	0.41
VEO	18	−0.65	−0.69	−0.61	<.0001
20% ASiR	24	−0.20	−0.23	−0.16	<.0001
40% ASiR	24	−0.35	−0.39	−0.31	<.0001
70% ASiR	24	−0.61	−0.65	−0.58	<.0001
FBP	24	−0.08	−0.12	−0.04	0.0011
VEO	24	−0.70	−0.74	−0.67	<.0001

**Table 3:** Table A3. Summary of estimated mean CNR by Recon and ${\text{CTDI}}_{\text{vol}}$. For example, VEO at ${\text{CTDI}}_{\text{vol}}$ 1 yielded a CNR of 0.81 (95% CI: 0.73−0.90), whereas FBP yielded a CNR of 0.16 (95% CI: 0.08−0.24) at the same dose

*Recon*	${\text{CTDI}}_{\text{vol}}$	*Estimated Mean CNR*	*95% LCL*	*95% UCL*
20% ASiR	1	0.19	0.10	0.27
40% ASiR	1	0.22	0.14	0.30
70% ASiR	1	0.29	0.21	0.37
FBP	1	0.16	0.08	0.24
VEO	1	0.81	0.73	0.90
20% ASiR	1.5	0.28	0.19	0.36
40% ASiR	1.5	0.32	0.23	0.40
70% ASiR	1.5	0.40	0.32	0.49
FBP	1.5	0.26	0.18	0.34
VEO	1.5	0.74	0.66	0.82
20% ASiR	3	0.32	0.23	0.40
40% ASiR	3	0.37	0.28	0.45
70% ASiR	3	0.48	0.40	0.56
FBP	3	0.28	0.19	0.36
VEO	3	0.89	0.80	0.97
20% ASiR	6	0.48	0.39	0.56
40% ASiR	6	0.55	0.47	0.64
70% ASiR	6	0.71	0.63	0.80
FBP	6	0.42	0.34	0.50
VEO	6	1.07	0.99	1.16
20% ASiR	12	0.70	0.61	0.78
40% ASiR	12	0.81	0.73	0.89
70% ASiR	12	1.06	0.98	1.14
FBP	12	0.61	0.53	0.69
VEO	12	1.37	1.29	1.45
20% ASiR	18	0.81	0.73	0.89
40% ASiR	18	0.94	0.86	1.03
70% ASiR	18	1.23	1.15	1.31
FBP	18	0.71	0.63	0.79
VEO	18	1.52	1.44	1.60
20% ASiR	24	0.90	0.82	0.98
40% ASiR	24	1.05	0.97	1.14
70% ASiR	24	1.38	1.30	1.46
FBP	24	0.79	0.71	0.87
VEO	24	1.58	1.50	1.67

**Table 4:** Table A4. Summary of estimated mean CNR difference between Recon/${\text{CTDI}}_{\text{vol}}$ combinations and the reference (VEO at ${\text{CTDI}}_{\text{vol}}$ of 1). A positive difference means higher CNR (better), a negative difference means lower CNR (worse). For example, 20% ASiR at ${\text{CTDI}}_{\text{vol}}$ of 1 yielded significantly worse CNR compared to VEO at the same dose (difference=−0.63, 95% CI: −0.74–−0.51, adjusted p‐value<0.0001). Dunnett's adjustment was used to control overall type 1 error rate at 5%

*Recon*	${\text{CTDI}}_{\text{vol}}$	*Estimated Mean CNR Difference*	*95% LCL*	*95% UCL*	*Adjusted p‐value*
20% ASiR	1	−0.63	−0.74	−0.51	<.0001
40% ASiR	1	−0.60	−0.71	−0.48	<.0001
70% ASiR	1	−0.53	−0.64	−0.41	<.0001
FBP	1	−0.65	−0.77	−0.54	<.0001
20% ASiR	1.5	−0.54	−0.65	−0.42	<.0001
40% ASiR	1.5	−0.50	−0.61	−0.38	<.0001
70% ASiR	1.5	−0.41	−0.53	−0.30	<.0001
FBP	1.5	−0.56	−0.67	−0.44	<.0001
VEO	1.5	−0.07	−0.19	0.04	0.98
20% ASiR	3	−0.50	−0.61	−0.38	<.0001
40% ASiR	3	−0.45	−0.56	−0.33	<.0001
70% ASiR	3	−0.33	−0.45	−0.22	<.0001
FBP	3	−0.54	−0.65	−0.42	<.0001
VEO	3	0.07	−0.04	0.19	0.99
20% ASiR	6	−0.34	−0.45	−0.22	<.0001
40% ASiR	6	−0.26	−0.38	−0.15	0.0003
70% ASiR	6	−0.10	−0.22	0.01	0.70
FBP	6	−0.39	−0.51	−0.28	<.0001
VEO	6	0.26	0.14	0.37	0.0004
20% ASiR	12	−0.12	−0.23	0.00	0.48
40% ASiR	12	0.00	−0.12	0.11	1.00
70% ASiR	12	0.24	0.13	0.36	0.0012
FBP	12	−0.21	−0.32	−0.09	0.01
VEO	12	0.55	0.44	0.67	<.0001
20% ASiR	18	−0.01	−0.12	0.11	1.00
40% ASiR	18	0.13	0.01	0.24	0.38
70% ASiR	18	0.42	0.30	0.53	<.0001
FBP	18	−0.10	−0.22	0.01	0.69
VEO	18	0.71	0.59	0.82	<.0001
20% ASiR	24	0.09	−0.03	0.20	0.90
40% ASiR	24	0.24	0.12	0.35	0.0018
70% ASiR	24	0.56	0.45	0.68	<.0001
FBP	24	−0.02	−0.14	0.09	1.00
VEO	24	0.77	0.65	0.88	<.0001

**Table 5:** Table A5. Summary of estimated ratio between algorithms with respect to MTF by setting. For example, MTF of 20% ASiR at setting of pitch 0.516, dose 0.89, and frequency 5.625 was 86% of FBP (95% CI: 45–163%). Estimated mean ratio higher than 1 means larger MTF than FBP, and lower than 1 means smaller MTF than FBP. MTF was transformed to the logarithmic scale prior to ANOVA analysis. Estimated differences on the logarithmic scale were back‐transformed as ratio to the raw scale as shown in the table. Dunnett's adjustment was used to control overall type 1 error rate at 5% for each model

*Pitch*	*Dose*	*Frequency*	*Ratio*	*Estimated Mean Ratio*	*95% LCL*	*95% UCL*	*Adjusted p‐value*
0.516	0.89	5.625	20% ASiR/FBP	0.86	0.45	1.63	0.97
0.516	0.89	5.625	40% ASiR/FBP	1.02	0.54	1.95	1.00
0.516	0.89	5.625	70% ASiR/FBP	1.14	0.60	2.17	0.99
0.516	0.89	5.625	MBIR/FBP	0.85	0.45	1.63	0.97
0.516	1.6	5.625	20% ASiR/FBP	0.97	0.54	1.75	1.00
0.516	1.6	5.625	40% ASiR/FBP	1.08	0.60	1.94	1.00
0.516	1.6	5.625	70% ASiR/FBP	1.18	0.66	2.13	0.95
0.516	1.6	5.625	MBIR/FBP	1.40	0.78	2.52	0.63
0.516	2.85	5.625	20% ASiR/FBP	1.32	0.75	2.31	0.73
0.516	2.85	5.625	40% ASiR/FBP	1.35	0.77	2.37	0.67
0.516	2.85	5.625	70% ASiR/FBP	2.18	1.25	3.82	0.02
0.516	2.85	5.625	MBIR/FBP	2.63	1.50	4.61	0.003
0.516	5.7	5.625	20% ASiR/FBP	1.48	0.76	2.89	0.60
0.516	5.7	5.625	40% ASiR/FBP	1.55	0.80	3.03	0.51
0.516	5.7	5.625	70% ASiR/FBP	2.15	1.10	4.19	0.09
0.516	5.7	5.625	MBIR/FBP	2.96	1.52	5.77	0.01
0.516	11.39	5.625	20% ASiR/FBP	1.08	0.62	1.88	1.00
0.516	11.39	5.625	40% ASiR/FBP	1.26	0.72	2.21	0.83
0.516	11.39	5.625	70% ASiR/FBP	1.50	0.86	2.62	0.42
0.516	11.39	5.625	MBIR/FBP	4.72	2.70	8.25	<.0001
0.516	18.7	5.625	20% ASiR/FBP	1.22	0.65	2.28	0.93
0.516	18.7	5.625	40% ASiR/FBP	1.49	0.80	2.79	0.54
0.516	18.7	5.625	70% ASiR/FBP	1.75	0.93	3.28	0.24
0.516	18.7	5.625	MBIR/FBP	8.11	4.33	15.20	<.0001
0.516	24.7	5.625	20% ASiR/FBP	0.92	0.49	1.71	1.00
0.516	24.7	5.625	40% ASiR/FBP	1.14	0.62	2.13	0.98
0.516	24.7	5.625	70% ASiR/FBP	1.22	0.66	2.28	0.92
0.516	24.7	5.625	MBIR/FBP	8.34	4.49	15.51	<.0001
0.984	0.89	5.625	20% ASiR/FBP	1.07	0.58	1.98	1.00
0.984	0.89	5.625	40% ASiR/FBP	0.99	0.54	1.83	1.00
0.984	0.89	5.625	70% ASiR/FBP	0.95	0.52	1.76	1.00
0.984	0.89	5.625	MBIR/FBP	1.25	0.68	2.31	0.88
0.984	1.6	5.625	20% ASiR/FBP	0.99	0.60	1.62	1.00
0.984	1.6	5.625	40% ASiR/FBP	0.94	0.57	1.54	1.00
0.984	1.6	5.625	70% ASiR/FBP	1.42	0.87	2.33	0.44
0.984	1.6	5.625	MBIR/FBP	1.53	0.93	2.52	0.27
0.984	2.85	5.625	20% ASiR/FBP	1.17	0.67	2.03	0.95
0.984	2.85	5.625	40% ASiR/FBP	1.42	0.82	2.48	0.53
0.984	2.85	5.625	70% ASiR/FBP	2.24	1.29	3.91	0.02
0.984	2.85	5.625	MBIR/FBP	3.06	1.76	5.33	0.0004
0.984	5.7	5.625	20% ASiR/FBP	1.26	0.73	2.20	0.83
0.984	5.7	5.625	40% ASiR/FBP	1.46	0.84	2.54	0.48
0.984	5.7	5.625	70% ASiR/FBP	2.30	1.32	4.00	0.01
0.984	5.7	5.625	MBIR/FBP	4.46	2.56	7.76	<.0001
0.984	11.39	5.625	20% ASiR/FBP	1.00	0.59	1.71	1.00
0.984	11.39	5.625	40% ASiR/FBP	1.39	0.81	2.37	0.57
0.984	11.39	5.625	70% ASiR/FBP	1.82	1.06	3.10	0.10
0.984	11.39	5.625	MBIR/FBP	5.47	3.21	9.34	<.0001
0.984	18.7	5.625	20% ASiR/FBP	1.01	0.55	1.85	1.00
0.984	18.7	5.625	40% ASiR/FBP	1.15	0.63	2.11	0.97
0.984	18.7	5.625	70% ASiR/FBP	1.31	0.71	2.39	0.80
0.984	18.7	5.625	MBIR/FBP	6.02	3.28	11.04	<.0001
0.984	24.7	5.625	20% ASiR/FBP	1.09	0.63	1.90	0.99
0.984	24.7	5.625	40% ASiR/FBP	1.30	0.75	2.27	0.75
0.984	24.7	5.625	70% ASiR/FBP	1.52	0.88	2.65	0.37
0.984	24.7	5.625	MBIR/FBP	6.78	3.90	11.77	<.0001
1.375	0.89	5.625	20%ASiR/FBP	0.81	0.48	1.38	0.85
1.375	0.89	5.625	40%ASiR/FBP	0.85	0.50	1.45	0.94
1.375	0.89	5.625	70%ASiR/FBP	0.89	0.53	1.52	0.98
1.375	0.89	5.625	MBIR/FBP	0.88	0.52	1.49	0.97
1.375	1.6	5.625	20%ASiR/FBP	1.31	0.73	2.36	0.78
1.375	1.6	5.625	40%ASiR/FBP	1.96	1.09	3.54	0.08
1.375	1.6	5.625	70%ASiR/FBP	2.42	1.34	4.35	0.01
1.375	1.6	5.625	MBIR/FBP	2.48	1.38	4.47	0.01
1.375	2.85	5.625	20%ASiR/FBP	1.19	0.62	2.28	0.96
1.375	2.85	5.625	40%ASiR/FBP	1.58	0.82	3.03	0.45
1.375	2.85	5.625	70%ASiR/FBP	1.39	0.73	2.67	0.71
1.375	2.85	5.625	MBIR/FBP	2.35	1.23	4.50	0.04
1.375	5.7	5.625	20%ASiR/FBP	1.29	0.75	2.20	0.76
1.375	5.7	5.625	40%ASiR/FBP	1.51	0.88	2.57	0.37
1.375	5.7	5.625	70%ASiR/FBP	2.12	1.24	3.62	0.02
1.375	5.7	5.625	MBIR/FBP	4.13	2.42	7.04	<.0001
1.375	11.39	5.625	20%ASiR/FBP	0.98	0.54	1.79	1.00
1.375	11.39	5.625	40%ASiR/FBP	0.99	0.54	1.81	1.00
1.375	11.39	5.625	70%ASiR/FBP	1.35	0.74	2.47	0.72
1.375	11.39	5.625	MBIR/FBP	4.71	2.58	8.60	<.0001
1.375	18.7	5.625	20%ASiR/FBP	1.05	0.56	1.96	1.00
1.375	18.7	5.625	40%ASiR/FBP	1.11	0.60	2.08	0.99
1.375	18.7	5.625	70%ASiR/FBP	1.56	0.84	2.92	0.43
1.375	18.7	5.625	MBIR/FBP	5.92	3.17	11.07	<.0001
1.375	24.7	5.625	20%ASiR/FBP	1.11	0.63	1.96	0.99
1.375	24.7	5.625	40%ASiR/FBP	1.01	0.57	1.78	1.00
1.375	24.7	5.625	70%ASiR/FBP	0.97	0.55	1.70	1.00
1.375	24.7	5.625	MBIR/FBP	6.40	3.63	11.29	<.0001

**Table 6:** Table A6. HU (SD) for the background and seven inserts of the Catphan 600 sensitometry module across reconstruction techniques and dose levels at 0.984 pitch

${\text{CTDI}}_{\text{vol}}$ *(mGy)*	*FBP*	*20% ASiR*	*40% ASiR*	*70%, ASiR*	*MBIR*	*FBP*	*20%, ASiR*	*40%, ASiR*	*70%, ASiR*	*MBIR*
	*Acrylic*					*Air*				
24	126 (2.5)	126 (2.5)	125 (2.4)	125 (2.4)	124 (1.8)	−952 (2.3)	−952 (2.3)	−952 (2.2)	−952 (2.1)	−957 (1.9)
19	125 (1.6)	125 (1.6)	125 (1.6)	125 (1.5)	126 (2.9)	−948 (1.9)	−948 (1.9)	−948 (1.7)	−948 (1.6)	−955 (1.7)
12	124 (2.6)	124 (2.6)	124 (2.4)	124 (2.3)	123(1.6)	−951 (3.4)	−951 (3.4)	−951 (3.2)	−951 (3.1)	−956 (3.3)
6	124 (5.6)	123 (5.4)	124 (5.2)	124 (5.0)	124 (4.8)	−949 (2.5)	−950 (2.6)	−949 (2.4)	−950 (2.4)	−953 (2.9)
3	125 (5.0)	124 (4.1)	125 (4.7)	125 (4.5)	124 (3.9)	−951 (3.6)	−951 (3.6)	−951 (3.5)	−951 (3.5)	−955 (2.3)
2	123 (6.6)	123 (6.6)	123 (6.2)	123 (5.9)	123 (4.6)	−953 (5.8)	−955 (5.0)	−954 (5.3)	−954 (5.0)	−952 (5.1)
1	124 (7.1)	124 (7.1)	124 (6.4)	124 (6.0)	120 (3.8)	−951 (8.7)	−954 (8.1)	−954 (8.4)	−955 (8.1)	−943 (7.2)
	*Delrin*					*PMP*				
24	340 (4.5)	341 (4.5)	340 (4.5)	340 (4.5)	340 (3.9)	−169 (1.9)	−169 (1.9)	−169 (1.8)	−169 (1.8)	−170 (2.0)
19	334 (3.3)	334 (3.3)	334 (3.1)	334 (3.0)	335 (2.0)	−168 (3.5)	−168 (3.6)	−168 (3.4)	−168 (3.3)	−172 (2.0)
12	339 (3.1)	339 (2.9)	339 (3.0)	339 (2.9)	340 (2.0)	−169 (3.3)	−169 (3.4)	−169 (3.1)	−169 (3.0)	−171 (2.4)
6	336 (5.1)	336 (4.5)	336 (4.9)	336 (4.7)	336 (3.8)	−168 (3.6)	−168 (3.7)	−168 (3.5)	−168 (3.5)	−171 (3.0)
3	336 (6.6)	335 (6.1)	336 (6.4)	336 (6.2)	336 (4.5)	−169 (3.7)	−169 (3.9)	−169 (3.6)	−170 (3.6)	−172 (2.4)
2	339 (8.5)	340 (8.6)	339 (8.0)	339 (7.6)	335 (6.5)	−163 (6.4)	−163 (6.4)	−163 (6.2)	−164 (6.1)	−166 (5.9)
1	339 (13.3)	338 (12.7)	340 (11.9)	340 (10.8)	327 (6.2)	−167 (10.1)	−169 (8.6)	−167 (9.4)	−168 (9.0)	−170 (7.7)
	*Teflon*					*LDPE*				
24	894 (4.0)	893 (4.1)	894 (3.9)	894 (3.8)	898 (1.9)	−79 (2.4)	−79 (2.5)	−79 (2.3)	−79 (2.2)	−83 (2.2)
19	893 (2.6)	893 (2.6)	893 (2.4)	893 (2.3)	896 (2.7)	−80 (2.1)	−80 (1.9)	−80 (2.0)	−80 (2.0)	−83 (1.8)
12	893 (4.4)	892 (4.3)	893 (4.2)	893 (4.0)	898 (2.6)	−79 (2.9)	−79 (2.9)	−79 (2.8)	−79 (2.7)	−82 (2.6)
6	894 (4.4)	895 (3.9)	894 (4.3)	894 (4.2)	894 (5.3)	−80 (4.2)	−80 (3.9)	−80 (3.6)	−80 (3.3)	−84 (2.9)
3	892(3.8)	892 (3.8)	893 (3.5)	893 (3.2)	894 (3.3)	−81 (6.1)	−81 (6.2)	−82 (5.6)	−81 (5.2)	−86 (3.7)
2	902 (6.7)	902 (6.9)	902 (6.4)	902 (6.2)	892 (8.5)	−80 (7.8)	−79 (7.7)	−79 (7.3)	−79 (6.9)	−86 (4.4)
1	899 (5.2)	900 (5.0)	900 (4.7)	900 (4.4)	880 (8.5)	−80 (8.4)	−80 (8.6)	−79 (8.1)	−79 (7.9)	−86 (5.1)
	*Background Material*					*Polystyrene*				
24	103 (1.0)	103 (0.9)	103 (1.0)	103 (1.0)	101(1.0)	−27 (3.3)	−27 (3.3)	−27 (3.1)	−27 (3.0)	−30 (2.3)
19	101 (0.8)	101 (0.8)	101 (0.9)	101 (0.8)	99 (0.6)	−28 (4.0)	−28 (3.8)	−28 (4.0)	−28 (3.9)	−29 (2.8)
12	102 (1.0)	103 (0.8)	102 (1.0)	102 (0.9)	100 (0.9)	−28 (1.9)	−27 (1.7)	−28 (1.6)	−28 (1.5)	−30 (1.4)
6	101 (1.2)	101 (1.1)	101 (1.1)	101 (1.1)	99 (0.9)	−31 (3.3)	−30 (3.0)	−31 (3.1)	−31 (3.0)	−33 (2.7)
3	101 (1.5)	101 (1.2)	101 (1.4)	101 (1.4)	98 (1.0)	−27 (4.4)	−26 (3.5)	−27 (4.3)	−27 (4.2)	−29 (4.5)
2	103 (1.6)	103 (1.6)	103 (1.6)	103 (1.6)	98 (1.7)	−23 (2.5)	−23 (2.4)	−23 (2.2)	−23 (2.1)	−28 (3.0)
1	102 (1.8)	102 (1.7)	102 (1.7)	102 (1.6)	95 (1.6)	−25 (7.7)	−25 (7.7)	−25 (7.1)	−25 (6.7)	−29 (5.6)

## Supporting information

Supplementary Material Files
