# Supplementary material for: Performance evaluation of iterative reconstruction algorithms for achieving CT radiation dose reduction — a phantom study
Source: J Appl Clin Med Phys. 2016 Mar 8;17(2):511–31. doi: 10.1120/jacmp.v17i2.5709 (PMC5875046; doi:10.1120/jacmp.v17i2.5709)
Supplement: Supplementary file 1 — Supplementary Material Files [file ACM2-17-511-s001.doc]

**SUPPLEMENTLA MATERIAL**

| **Table S1A. Summary of estimated mean noise by recon and CTDIvol. Noise was transformed to the logarithmic scale before being analyzed by linear mixed model. Interpretation: For example, VEO at CTDIvol 1 yielded a mean noise of 2.68 (95% CI: 2.65-2.70) whereas FBP at CTDIvol of 1 yielded a mean noise of 4.20 (95% CI: 4.17-4.22).** | | | | |
| --- | --- | --- | --- | --- |
| **Recon** | **CTDIvol** | **Estimated Mean Noise (logarithmic scale)** | **95% LCL** | **95% UCL** |
| **20% ASiR** | **1** | 4.06 | 4.03 | 4.09 |
| **40% ASiR** | **1** | 3.91 | 3.88 | 3.93 |
| **70% ASiR** | **1** | 3.64 | 3.61 | 3.66 |
| **FBP** | **1** | 4.20 | 4.17 | 4.22 |
| **VEO** | **1** | 2.68 | 2.65 | 2.70 |
| **20% ASiR** | **1.5** | 3.82 | 3.80 | 3.85 |
| **40% ASiR** | **1.5** | 3.67 | 3.64 | 3.70 |
| **70% ASiR** | **1.5** | 3.40 | 3.38 | 3.43 |
| **FBP** | **1.5** | 3.96 | 3.94 | 3.99 |
| **VEO** | **1.5** | 2.58 | 2.56 | 2.61 |
| **20% ASiR** | **3** | 3.49 | 3.46 | 3.52 |
| **40% ASiR** | **3** | 3.34 | 3.31 | 3.36 |
| **70% ASiR** | **3** | 3.07 | 3.04 | 3.09 |
| **FBP** | **3** | 3.63 | 3.60 | 3.66 |
| **VEO** | **3** | 2.46 | 2.43 | 2.48 |
| **20% ASiR** | **6** | 3.17 | 3.14 | 3.19 |
| **40% ASiR** | **6** | 3.01 | 2.98 | 3.04 |
| **70% ASiR** | **6** | 2.74 | 2.72 | 2.77 |
| **FBP** | **6** | 3.30 | 3.28 | 3.33 |
| **VEO** | **6** | 2.32 | 2.29 | 2.34 |
| **20% ASiR** | **12** | 2.83 | 2.80 | 2.85 |
| **40% ASiR** | **12** | 2.67 | 2.64 | 2.70 |
| **70% ASiR** | **12** | 2.40 | 2.37 | 2.43 |
| **FBP** | **12** | 2.97 | 2.94 | 2.99 |
| **VEO** | **12** | 2.20 | 2.18 | 2.23 |
| **20% ASiR** | **18** | 2.58 | 2.55 | 2.61 |
| **40% ASiR** | **18** | 2.43 | 2.40 | 2.45 |
| **70% ASiR** | **18** | 2.16 | 2.13 | 2.18 |
| **FBP** | **18** | 2.72 | 2.69 | 2.74 |
| **VEO** | **18** | 2.03 | 2.00 | 2.06 |
| **20% ASiR** | **24** | 2.48 | 2.45 | 2.51 |
| **40% ASiR** | **24** | 2.33 | 2.30 | 2.35 |
| **70% ASiR** | **24** | 2.06 | 2.04 | 2.09 |
| **FBP** | **24** | 2.60 | 2.57 | 2.62 |
| **VEO** | **24** | 1.97 | 1.95 | 2.00 |

| **Table S1B. Summary of estimated noise difference between Recon/CTDIvol combinations and the reference (VEO at CTDIvol of 1). Estimates were based on linear mixed model and Dunnett’s adjustment was used to control overall type I error rate at 5%. A positive difference means higher noise (worse), and a negative means lower noise (better). For example, 20% ASiR at CTDIvol of 1 yielded significantly higher noise than VEO at CTDIvol 1 (difference = 1.38, 95% CI: 1.35-1.42, adjusted p-value < 0.0001).** | | | | | |
| --- | --- | --- | --- | --- | --- |
| **Recon** | **CTDIvol** | **Estimated Mean Noise Difference (logarithmic scale)** | **95% LCL** | **95% UCL** | **Adjusted P-value** |
| **20% ASiR** | **1** | 1.38 | 1.35 | 1.42 | <.0001 |
| **40% ASiR** | **1** | 1.23 | 1.19 | 1.27 | <.0001 |
| **70% ASiR** | **1** | 0.96 | 0.92 | 1.00 | <.0001 |
| **FBP** | **1** | 1.52 | 1.48 | 1.56 | <.0001 |
| **20% ASiR** | **1.5** | 1.15 | 1.11 | 1.18 | <.0001 |
| **40% ASiR** | **1.5** | 0.99 | 0.96 | 1.03 | <.0001 |
| **70% ASiR** | **1.5** | 0.73 | 0.69 | 0.76 | <.0001 |
| **FBP** | **1.5** | 1.29 | 1.25 | 1.32 | <.0001 |
| **VEO** | **1.5** | -0.09 | -0.13 | -0.06 | <.0001 |
| **20% ASiR** | **3** | 0.81 | 0.78 | 0.85 | <.0001 |
| **40% ASiR** | **3** | 0.66 | 0.62 | 0.70 | <.0001 |
| **70% ASiR** | **3** | 0.39 | 0.35 | 0.43 | <.0001 |
| **FBP** | **3** | 0.95 | 0.91 | 0.99 | <.0001 |
| **VEO** | **3** | -0.22 | -0.26 | -0.18 | <.0001 |
| **20% ASiR** | **6** | 0.49 | 0.45 | 0.53 | <.0001 |
| **40% ASiR** | **6** | 0.33 | 0.30 | 0.37 | <.0001 |
| **70% ASiR** | **6** | 0.06 | 0.03 | 0.10 | 0.02 |
| **FBP** | **6** | 0.63 | 0.59 | 0.66 | <.0001 |
| **VEO** | **6** | -0.36 | -0.40 | -0.32 | <.0001 |
| **20% ASiR** | **12** | 0.15 | 0.11 | 0.19 | <.0001 |
| **40% ASiR** | **12** | -0.01 | -0.04 | 0.03 | 1.00 |
| **70% ASiR** | **12** | -0.28 | -0.31 | -0.24 | <.0001 |
| **FBP** | **12** | 0.29 | 0.25 | 0.32 | <.0001 |
| **VEO** | **12** | -0.48 | -0.51 | -0.44 | <.0001 |
| **20% ASiR** | **18** | -0.10 | -0.13 | -0.06 | <.0001 |
| **40% ASiR** | **18** | -0.25 | -0.29 | -0.22 | <.0001 |
| **70% ASiR** | **18** | -0.52 | -0.56 | -0.48 | <.0001 |
| **FBP** | **18** | 0.04 | 0.003 | 0.08 | 0.41 |
| **VEO** | **18** | -0.65 | -0.69 | -0.61 | <.0001 |
| **20% ASiR** | **24** | -0.20 | -0.23 | -0.16 | <.0001 |
| **40% ASiR** | **24** | -0.35 | -0.39 | -0.31 | <.0001 |
| **70% ASiR** | **24** | -0.61 | -0.65 | -0.58 | <.0001 |
| **FBP** | **24** | -0.08 | -0.12 | -0.04 | 0.0011 |
| **VEO** | **24** | -0.70 | -0.74 | -0.67 | <.0001 |

| **Table S2A. Summary of estimated mean CNR by recon and CTDIvol. Interpretation: for example, VEO at CTDIvol 1 yielded a CNR of 0.81 (95% CI: 0.73-0.90), whereas FBP yielded a CNR of 0.16 (95% CI: 0.08-0.24) at the same dose.** | | | | |
| --- | --- | --- | --- | --- |
| **Recon** | **CTDIvol** | **Estimated Mean CNR** | **95% LCL** | **95% UCL** |
| **20% ASiR** | **1** | 0.19 | 0.10 | 0.27 |
| **40% ASiR** | **1** | 0.22 | 0.14 | 0.30 |
| **70% ASiR** | **1** | 0.29 | 0.21 | 0.37 |
| **FBP** | **1** | 0.16 | 0.08 | 0.24 |
| **VEO** | **1** | 0.81 | 0.73 | 0.90 |
| **20% ASiR** | **1.5** | 0.28 | 0.19 | 0.36 |
| **40% ASiR** | **1.5** | 0.32 | 0.23 | 0.40 |
| **70% ASiR** | **1.5** | 0.40 | 0.32 | 0.49 |
| **FBP** | **1.5** | 0.26 | 0.18 | 0.34 |
| **VEO** | **1.5** | 0.74 | 0.66 | 0.82 |
| **20% ASiR** | **3** | 0.32 | 0.23 | 0.40 |
| **40% ASiR** | **3** | 0.37 | 0.28 | 0.45 |
| **70% ASiR** | **3** | 0.48 | 0.40 | 0.56 |
| **FBP** | **3** | 0.28 | 0.19 | 0.36 |
| **VEO** | **3** | 0.89 | 0.80 | 0.97 |
| **20% ASiR** | **6** | 0.48 | 0.39 | 0.56 |
| **40% ASiR** | **6** | 0.55 | 0.47 | 0.64 |
| **70% ASiR** | **6** | 0.71 | 0.63 | 0.80 |
| **FBP** | **6** | 0.42 | 0.34 | 0.50 |
| **VEO** | **6** | 1.07 | 0.99 | 1.16 |
| **20% ASiR** | **12** | 0.70 | 0.61 | 0.78 |
| **40% ASiR** | **12** | 0.81 | 0.73 | 0.89 |
| **70% ASiR** | **12** | 1.06 | 0.98 | 1.14 |
| **FBP** | **12** | 0.61 | 0.53 | 0.69 |
| **VEO** | **12** | 1.37 | 1.29 | 1.45 |
| **20% ASiR** | **18** | 0.81 | 0.73 | 0.89 |
| **40% ASiR** | **18** | 0.94 | 0.86 | 1.03 |
| **70% ASiR** | **18** | 1.23 | 1.15 | 1.31 |
| **FBP** | **18** | 0.71 | 0.63 | 0.79 |
| **VEO** | **18** | 1.52 | 1.44 | 1.60 |
| **20% ASiR** | **24** | 0.90 | 0.82 | 0.98 |
| **40% ASiR** | **24** | 1.05 | 0.97 | 1.14 |
| **70% ASiR** | **24** | 1.38 | 1.30 | 1.46 |
| **FBP** | **24** | 0.79 | 0.71 | 0.87 |
| **VEO** | **24** | 1.58 | 1.50 | 1.67 |

| **Table S2B. Summary of estimated mean CNR difference between recon/CTDIvol combinations and the reference (VEO at CTDIvol of 1). A positive difference means higher CNR (better), a negative difference means lower CNR (worse). For example, 20% ASiR at CTDIvol of 1 yielded significantly worse CNR compared to VEO at the same dose (difference = -0.63, 95% CI: -0.74 – -0.51, adjusted p-value < 0.0001). Dunnett’s adjustment was used to control overall type I error rate at 5%.** | | | | | | | | | | | | |  |
| --- | --- | --- | --- | --- | --- | --- | --- | --- | --- | --- | --- | --- | --- |
| **Recon** | | **CTDIvol** | | **Estimated Mean CNR Difference** | | **95% LCL** | | **95% UCL** | | | **Adjusted P-value** | |  |
| **20% ASiR** | | **1** | | -0.63 | | -0.74 | | -0.51 | | | <.0001 | |  |
| **40% ASiR** | | **1** | | -0.60 | | -0.71 | | -0.48 | | | <.0001 | |  |
| **70% ASiR** | | **1** | | -0.53 | | -0.64 | | -0.41 | | | <.0001 | |  |
| **FBP** | | **1** | | -0.65 | | -0.77 | | -0.54 | | | <.0001 | |  |
| **20% ASiR** | | **1.5** | | -0.54 | | -0.65 | | -0.42 | | | <.0001 | |  |
| **40% ASiR** | | **1.5** | | -0.50 | | -0.61 | | -0.38 | | | <.0001 | |  |
| **70% ASiR** | | **1.5** | | -0.41 | | -0.53 | | -0.30 | | | <.0001 | |  |
| **FBP** | | **1.5** | | -0.56 | | -0.67 | | -0.44 | | | <.0001 | |  |
| **VEO** | | **1.5** | | -0.07 | | -0.19 | | 0.04 | | | 0.98 | |  |
| **20% ASiR** | | **3** | | -0.50 | | -0.61 | | -0.38 | | | <.0001 | |  |
| **40% ASiR** | | **3** | | -0.45 | | -0.56 | | -0.33 | | | <.0001 | |  |
| **70% ASiR** | | **3** | | -0.33 | | -0.45 | | -0.22 | | | <.0001 | |  |
| **FBP** | | **3** | | -0.54 | | -0.65 | | -0.42 | | | <.0001 | |  |
| **VEO** | | **3** | | 0.07 | | -0.04 | | 0.19 | | | 0.99 | |  |
| **20% ASiR** | | **6** | | -0.34 | | -0.45 | | -0.22 | | | <.0001 | |  |
| **40% ASiR** | | **6** | | -0.26 | | -0.38 | | -0.15 | | | 0.0003 | |  |
| **70% ASiR** | | **6** | | -0.10 | | -0.22 | | 0.01 | | | 0.70 | |  |
| **FBP** | | **6** | | -0.39 | | -0.51 | | -0.28 | | | <.0001 | |  |
| **VEO** | | **6** | | 0.26 | | 0.14 | | 0.37 | | | 0.0004 | |  |
| **20% ASiR** | | **12** | | -0.12 | | -0.23 | | 0.00 | | | 0.48 | |  |
| **40% ASiR** | | **12** | | 0.00 | | -0.12 | | 0.11 | | | 1.00 | |  |
| **70% ASiR** | | **12** | | 0.24 | | 0.13 | | 0.36 | | | 0.0012 | |  |
| **FBP** | | **12** | | -0.21 | | -0.32 | | -0.09 | | | 0.01 | |  |
| **VEO** | | **12** | | 0.55 | | 0.44 | | 0.67 | | | <.0001 | |  |
| **20% ASiR** | | **18** | | -0.01 | | -0.12 | | 0.11 | | | 1.00 | |  |
| **40% ASiR** | | **18** | | 0.13 | | 0.01 | | 0.24 | | | 0.38 | |  |
| **70% ASiR** | | **18** | | 0.42 | | 0.30 | | 0.53 | | | <.0001 | |  |
| **FBP** | | **18** | | -0.10 | | -0.22 | | 0.01 | | | 0.69 | |  |
| **VEO** | | **18** | | 0.71 | | 0.59 | | 0.82 | | | <.0001 | |  |
| **20% ASiR** | | **24** | | 0.09 | | -0.03 | | 0.20 | | | 0.90 | |  |
| **40% ASiR** | | **24** | | 0.24 | | 0.12 | | 0.35 | | | 0.0018 | |  |
| **70% ASiR** | | **24** | | 0.56 | | 0.45 | | 0.68 | | | <.0001 | |  |
| **FBP** | | **24** | | -0.02 | | -0.14 | | 0.09 | | | 1.00 | |  |
| **VEO** | | **24** | | 0.77 | | 0.65 | | 0.88 | | | <.0001 | |  |
| **Table S3. Summary of estimated ratio between algorithms with respect to MTF by setting. For example, MTF of 20% ASiR at setting of pitch 0.516, dose 0.89, and frequency 5.625 was 86% of FBP (95% CI: 45 – 163%). Estimated mean ratio higher than 1 means larger MTF than FBP, and lower than 1 means smaller MTF than FBP. MTF was transformed to the logarithmic scale prior to ANOVA analysis. Estimated differences on the logarithmic scale were back-transformed as ratio to the raw scale as shown in the table. Dunnett’s adjustment was used to control overall type I error rate at 5% for each model.** | | | | | | | | | | | | | |
| **Pitch** | **Dose** | | **Frequency** | | **Ratio** | | **Estimated Mean Ratio** | | **95% LCL** | **95% UCL** | | **Adjusted P-value** | |
| **0.516** | 0.89 | | 5.625 | | 20% ASiR/FBP | | 0.86 | | 0.45 | 1.63 | | 0.97 | |
| **0.516** | 0.89 | | 5.625 | | 40% ASiR/FBP | | 1.02 | | 0.54 | 1.95 | | 1.00 | |
| **0.516** | 0.89 | | 5.625 | | 70% ASiR/FBP | | 1.14 | | 0.60 | 2.17 | | 0.99 | |
| **0.516** | 0.89 | | 5.625 | | MBIR/FBP | | 0.85 | | 0.45 | 1.63 | | 0.97 | |
| **0.516** | 1.6 | | 5.625 | | 20% ASiR/FBP | | 0.97 | | 0.54 | 1.75 | | 1.00 | |
| **0.516** | 1.6 | | 5.625 | | 40% ASiR/FBP | | 1.08 | | 0.60 | 1.94 | | 1.00 | |
| **0.516** | 1.6 | | 5.625 | | 70% ASiR/FBP | | 1.18 | | 0.66 | 2.13 | | 0.95 | |
| **0.516** | 1.6 | | 5.625 | | MBIR/FBP | | 1.40 | | 0.78 | 2.52 | | 0.63 | |
| **0.516** | 2.85 | | 5.625 | | 20% ASiR/FBP | | 1.32 | | 0.75 | 2.31 | | 0.73 | |
| **0.516** | 2.85 | | 5.625 | | 40% ASiR/FBP | | 1.35 | | 0.77 | 2.37 | | 0.67 | |
| **0.516** | 2.85 | | 5.625 | | 70% ASiR/FBP | | 2.18 | | 1.25 | 3.82 | | 0.02 | |
| **0.516** | 2.85 | | 5.625 | | MBIR/FBP | | 2.63 | | 1.50 | 4.61 | | 0.003 | |
| **0.516** | 5.7 | | 5.625 | | 20% ASiR/FBP | | 1.48 | | 0.76 | 2.89 | | 0.60 | |
| **0.516** | 5.7 | | 5.625 | | 40% ASiR/FBP | | 1.55 | | 0.80 | 3.03 | | 0.51 | |
| **0.516** | 5.7 | | 5.625 | | 70% ASiR/FBP | | 2.15 | | 1.10 | 4.19 | | 0.09 | |
| **0.516** | 5.7 | | 5.625 | | MBIR/FBP | | 2.96 | | 1.52 | 5.77 | | 0.01 | |
| **0.516** | 11.39 | | 5.625 | | 20% ASiR/FBP | | 1.08 | | 0.62 | 1.88 | | 1.00 | |
| **0.516** | 11.39 | | 5.625 | | 40% ASiR/FBP | | 1.26 | | 0.72 | 2.21 | | 0.83 | |
| **0.516** | 11.39 | | 5.625 | | 70% ASiR/FBP | | 1.50 | | 0.86 | 2.62 | | 0.42 | |
| **0.516** | 11.39 | | 5.625 | | MBIR/FBP | | 4.72 | | 2.70 | 8.25 | | <.0001 | |
| **0.516** | 18.7 | | 5.625 | | 20% ASiR/FBP | | 1.22 | | 0.65 | 2.28 | | 0.93 | |
| **0.516** | 18.7 | | 5.625 | | 40% ASiR/FBP | | 1.49 | | 0.80 | 2.79 | | 0.54 | |
| **0.516** | 18.7 | | 5.625 | | 70% ASiR/FBP | | 1.75 | | 0.93 | 3.28 | | 0.24 | |
| **0.516** | 18.7 | | 5.625 | | MBIR/FBP | | 8.11 | | 4.33 | 15.20 | | <.0001 | |
| **0.516** | 24.7 | | 5.625 | | 20% ASiR/FBP | | 0.92 | | 0.49 | 1.71 | | 1.00 | |
| **0.516** | 24.7 | | 5.625 | | 40% ASiR/FBP | | 1.14 | | 0.62 | 2.13 | | 0.98 | |
| **0.516** | 24.7 | | 5.625 | | 70% ASiR/FBP | | 1.22 | | 0.66 | 2.28 | | 0.92 | |
| **0.516** | 24.7 | | 5.625 | | MBIR/FBP | | 8.34 | | 4.49 | 15.51 | | <.0001 | |
| **0.984** | 0.89 | | 5.625 | | 20% ASiR/FBP | | 1.07 | | 0.58 | 1.98 | | 1.00 | |
| **0.984** | 0.89 | | 5.625 | | 40% ASiR/FBP | | 0.99 | | 0.54 | 1.83 | | 1.00 | |
| **0.984** | 0.89 | | 5.625 | | 70% ASiR/FBP | | 0.95 | | 0.52 | 1.76 | | 1.00 | |
| **0.984** | 0.89 | | 5.625 | | MBIR/FBP | | 1.25 | | 0.68 | 2.31 | | 0.88 | |
| **0.984** | 1.6 | | 5.625 | | 20% ASiR/FBP | | 0.99 | | 0.60 | 1.62 | | 1.00 | |
| **0.984** | 1.6 | | 5.625 | | 40% ASiR/FBP | | 0.94 | | 0.57 | 1.54 | | 1.00 | |
| **0.984** | 1.6 | | 5.625 | | 70% ASiR/FBP | | 1.42 | | 0.87 | 2.33 | | 0.44 | |
| **0.984** | 1.6 | | 5.625 | | MBIR/FBP | | 1.53 | | 0.93 | 2.52 | | 0.27 | |
| **0.984** | 2.85 | | 5.625 | | 20% ASiR/FBP | | 1.17 | | 0.67 | 2.03 | | 0.95 | |
| **0.984** | 2.85 | | 5.625 | | 40% ASiR/FBP | | 1.42 | | 0.82 | 2.48 | | 0.53 | |
| **0.984** | 2.85 | | 5.625 | | 70% ASiR/FBP | | 2.24 | | 1.29 | 3.91 | | 0.02 | |
| **0.984** | 2.85 | | 5.625 | | MBIR/FBP | | 3.06 | | 1.76 | 5.33 | | 0.0004 | |
| **0.984** | 5.7 | | 5.625 | | 20% ASiR/FBP | | 1.26 | | 0.73 | 2.20 | | 0.83 | |
| **0.984** | 5.7 | | 5.625 | | 40% ASiR/FBP | | 1.46 | | 0.84 | 2.54 | | 0.48 | |
| **0.984** | 5.7 | | 5.625 | | 70% ASiR/FBP | | 2.30 | | 1.32 | 4.00 | | 0.01 | |
| **0.984** | 5.7 | | 5.625 | | MBIR/FBP | | 4.46 | | 2.56 | 7.76 | | <.0001 | |
| **0.984** | 11.39 | | 5.625 | | 20% ASiR/FBP | | 1.00 | | 0.59 | 1.71 | | 1.00 | |
| **0.984** | 11.39 | | 5.625 | | 40% ASiR/FBP | | 1.39 | | 0.81 | 2.37 | | 0.57 | |
| **0.984** | 11.39 | | 5.625 | | 70% ASiR/FBP | | 1.82 | | 1.06 | 3.10 | | 0.10 | |
| **0.984** | 11.39 | | 5.625 | | MBIR/FBP | | 5.47 | | 3.21 | 9.34 | | <.0001 | |
| **0.984** | 18.7 | | 5.625 | | 20% ASiR/FBP | | 1.01 | | 0.55 | 1.85 | | 1.00 | |
| **0.984** | 18.7 | | 5.625 | | 40% ASiR/FBP | | 1.15 | | 0.63 | 2.11 | | 0.97 | |
| **0.984** | 18.7 | | 5.625 | | 70% ASiR/FBP | | 1.31 | | 0.71 | 2.39 | | 0.80 | |
| **0.984** | 18.7 | | 5.625 | | MBIR/FBP | | 6.02 | | 3.28 | 11.04 | | <.0001 | |
| **0.984** | 24.7 | | 5.625 | | 20% ASiR/FBP | | 1.09 | | 0.63 | 1.90 | | 0.99 | |
| **0.984** | 24.7 | | 5.625 | | 40% ASiR/FBP | | 1.30 | | 0.75 | 2.27 | | 0.75 | |
| **0.984** | 24.7 | | 5.625 | | 70% ASiR/FBP | | 1.52 | | 0.88 | 2.65 | | 0.37 | |
| **0.984** | 24.7 | | 5.625 | | MBIR/FBP | | 6.78 | | 3.90 | 11.77 | | <.0001 | |
| **1.375** | 0.89 | | 5.625 | | 20% ASiR/FBP | | 0.81 | | 0.48 | 1.38 | | 0.85 | |
| **1.375** | 0.89 | | 5.625 | | 40% ASiR/FBP | | 0.85 | | 0.50 | 1.45 | | 0.94 | |
| **1.375** | 0.89 | | 5.625 | | 70% ASiR/FBP | | 0.89 | | 0.53 | 1.52 | | 0.98 | |
| **1.375** | 0.89 | | 5.625 | | MBIR/FBP | | 0.88 | | 0.52 | 1.49 | | 0.97 | |
| **1.375** | 1.6 | | 5.625 | | 20% ASiR/FBP | | 1.31 | | 0.73 | 2.36 | | 0.78 | |
| **1.375** | 1.6 | | 5.625 | | 40% ASiR/FBP | | 1.96 | | 1.09 | 3.54 | | 0.08 | |
| **1.375** | 1.6 | | 5.625 | | 70% ASiR/FBP | | 2.42 | | 1.34 | 4.35 | | 0.01 | |
| **1.375** | 1.6 | | 5.625 | | MBIR/FBP | | 2.48 | | 1.38 | 4.47 | | 0.01 | |
| **1.375** | 2.85 | | 5.625 | | 20% ASiR/FBP | | 1.19 | | 0.62 | 2.28 | | 0.96 | |
| **1.375** | 2.85 | | 5.625 | | 40% ASiR/FBP | | 1.58 | | 0.82 | 3.03 | | 0.45 | |
| **1.375** | 2.85 | | 5.625 | | 70% ASiR/FBP | | 1.39 | | 0.73 | 2.67 | | 0.71 | |
| **1.375** | 2.85 | | 5.625 | | MBIR/FBP | | 2.35 | | 1.23 | 4.50 | | 0.04 | |
| **1.375** | 5.7 | | 5.625 | | 20% ASiR/FBP | | 1.29 | | 0.75 | 2.20 | | 0.76 | |
| **1.375** | 5.7 | | 5.625 | | 40% ASiR/FBP | | 1.51 | | 0.88 | 2.57 | | 0.37 | |
| **1.375** | 5.7 | | 5.625 | | 70% ASiR/FBP | | 2.12 | | 1.24 | 3.62 | | 0.02 | |
| **1.375** | 5.7 | | 5.625 | | MBIR/FBP | | 4.13 | | 2.42 | 7.04 | | <.0001 | |
| **1.375** | 11.39 | | 5.625 | | 20% ASiR/FBP | | 0.98 | | 0.54 | 1.79 | | 1.00 | |
| **1.375** | 11.39 | | 5.625 | | 40% ASiR/FBP | | 0.99 | | 0.54 | 1.81 | | 1.00 | |
| **1.375** | 11.39 | | 5.625 | | 70% ASiR/FBP | | 1.35 | | 0.74 | 2.47 | | 0.72 | |
| **1.375** | 11.39 | | 5.625 | | MBIR/FBP | | 4.71 | | 2.58 | 8.60 | | <.0001 | |
| **1.375** | 18.7 | | 5.625 | | 20% ASiR/FBP | | 1.05 | | 0.56 | 1.96 | | 1.00 | |
| **1.375** | 18.7 | | 5.625 | | 40% ASiR/FBP | | 1.11 | | 0.60 | 2.08 | | 0.99 | |
| **1.375** | 18.7 | | 5.625 | | 70% ASiR/FBP | | 1.56 | | 0.84 | 2.92 | | 0.43 | |
| **1.375** | 18.7 | | 5.625 | | MBIR/FBP | | 5.92 | | 3.17 | 11.07 | | <.0001 | |
| **1.375** | 24.7 | | 5.625 | | 20% ASiR/FBP | | 1.11 | | 0.63 | 1.96 | | 0.99 | |
| **1.375** | 24.7 | | 5.625 | | 40% ASiR/FBP | | 1.01 | | 0.57 | 1.78 | | 1.00 | |
| **1.375** | 24.7 | | 5.625 | | 70% ASiR/FBP | | 0.97 | | 0.55 | 1.70 | | 1.00 | |
| **1.375** | 24.7 | | 5.625 | | MBIR/FBP | | 6.40 | | 3.63 | 11.29 | | <.0001 | |

| TABLE S4. HU (SD) for the background and 7 inserts of the Catphan 600  sensitometry module across reconstruction techniques and dose levels at 0.984 pitch. | | | | | | | | | | | | |
| --- | --- | --- | --- | --- | --- | --- | --- | --- | --- | --- | --- | --- |
|  |  |  |  |  |  |  |  |  |  |  |  |  |
| **CTDI_vol_ (mGy)** |  | **FBP** | **20% ASiR** | **40% ASiR** | **70% ASiR** | **MBIR** |  | **FBP** | **20% ASiR** | **40% ASiR** | **70% ASiR** | **MBIR** |
|  |  |  |  |  |  |  |  |  |  |  |  |
|  |  | **Acrylic** | | | | |  | **Air** | | | | |
|  |  |  |  |  |  |  |  |  |  |  |  |  |
| 24 |  | 126 (2.5) | 126 (2.5) | 125 (2.4) | 125 (2.4) | 124 (1.8) |  | -952 (2.3) | -952 (2.3) | -952 (2.2) | -952 (2.1) | -957 (1.9) |
| 19 |  | 125 (1.6) | 125 (1.6) | 125 (1.6) | 125 (1.5) | 126 (2.9) |  | -948 (1.9) | -948 (1.9) | -948 (1.7) | -948 (1.6) | -955 (1.7) |
| 12 |  | 124 (2.6) | 124 (2.6) | 124 (2.4) | 124 (2.3) | 123 (1.6) |  | -951 (3.4) | -951 (3.4) | -951 (3.2) | -951 (3.1) | -956 (3.3) |
| 6 |  | 124 (5.6) | 123 (5.4) | 124 (5.2) | 124 (5.0) | 124 (4.8) |  | -949 (2.5) | -950 (2.6) | -949 (2.4) | -950 (2.4) | -953 (2.9) |
| 3 |  | 125 (5.0) | 124 (4.1) | 125 (4.7) | 125 (4.5) | 124 (3.9) |  | -951 (3.6) | -951 (3.6) | -951 (3.5) | -951 (3.5) | -955 (2.3) |
| 2 |  | 123 (6.6) | 123 (6.6) | 123 (6.2) | 123 (5.9) | 123 (4.6) |  | -953 (5.8) | -955 (5.0) | -954 (5.3) | -954 (5.0) | -952 (5.1) |
| 1 |  | 124 (7.1) | 124 (7.1) | 124 (6.4) | 124 (6.0) | 120 (3.8) |  | -951 (8.7) | -954 (8.1) | -954 (8.4) | -955 (8.1) | -943 (7.2) |
|  |  |  |  |  |  |  |  |  |  |  |  |  |
|  |  | **Delrin** | | | | |  | **PMP** | | | | |
|  |  |  |  |  |  |  |  |  |  |  |  |  |
| 24 |  | 340 (4.5) | 341 (4.5) | 340 (4.5) | 340 (4.5) | 340 (3.9) |  | -169 (1.9) | -169 (1.9) | -169 (1.8) | -169 (1.8) | -170 (2.0) |
| 19 |  | 334 (3.3) | 334 (3.3) | 334 (3.1) | 334 (3.0) | 335 (2.0) |  | -168 (3.5) | -168 (3.6) | -168 (3.4) | -168 (3.3) | -172 (2.0) |
| 12 |  | 339 (3.1) | 339 (2.9) | 339 (3.0) | 339 (2.9) | 340 (2.0) |  | -169 (3.3) | -169 (3.4) | -169 (3.1) | -169 (3.0) | -171 (2.4) |
| 6 |  | 336 (5.1) | 336 (4.5) | 336 (4.9) | 336 (4.7) | 336 (3.8) |  | -168 (3.6) | -168 (3.7) | -168 (3.5) | -168 (3.5) | -171 (3.0) |
| 3 |  | 336 (6.6) | 335 (6.1) | 336 (6.4) | 336 (6.2) | 336 (4.5) |  | -169 (3.7) | -169 (3.9) | -169 (3.6) | -170 (3.6) | -172 (2.4) |
| 2 |  | 339 (8.5) | 340 (8.6) | 339 (8.0) | 339 (7.6) | 335 (6.5) |  | -163 (6.4) | -163 (6.4) | -163 (6.2) | -164 (6.1) | -166 (5.9) |
| 1 |  | 339 (13.3) | 338 (12.7) | 340 (11.9) | 340 (10.8) | 327 (6.2) |  | -167 (10.1) | -169 (8.6) | -167 (9.4) | -168 (9.0) | -170 (7.7) |
|  |  |  |  |  |  |  |  |  |  |  |  |  |
|  |  | **Teflon** | | | | |  | **LDPE** | | | | |
|  |  |  |  |  |  |  |  |  |  |  |  |  |
| 24 |  | 894 (4.0) | 893 (4.1) | 894 (3.9) | 894 (3.8) | 898 (1.9) |  | -79 (2.4) | -79 (2.5) | -79 (2.3) | -79 (2.2) | -83 (2.2) |
| 19 |  | 893 (2.6) | 893 (2.6) | 893 (2.4) | 893 (2.3) | 896 (2.7) |  | -80 (2.1) | -80 (1.9) | -80 (2.0) | -80 (2.0) | -83 (1.8) |
| 12 |  | 893 (4.4) | 892 (4.3) | 893 (4.2) | 893 (4.0) | 898 (2.6) |  | -79 (2.9) | -79 (2.9) | -79 (2.8) | -79 (2.7) | -82 (2.6) |
| 6 |  | 894 (4.4) | 895 (3.9) | 894 (4.3) | 894 (4.2) | 894 (5.3) |  | -80 (4.2) | -80 (3.9) | -80 (3.6) | -80 (3.3) | -84 (2.9) |
| 3 |  | 892 (3.8) | 892 (3.8) | 893 (3.5) | 893 (3.2) | 894 (3.3) |  | -81 (6.1) | -81 (6.2) | -82 (5.6) | -81 (5.2) | -86 (3.7) |
| 2 |  | 902 (6.7) | 902 (6.9) | 902 (6.4) | 902 (6.2) | 892 (8.5) |  | -80 (7.8) | -79 (7.7) | -79 (7.3) | -79 (6.9) | -86 (4.4) |
| 1 |  | 899 (5.2) | 900 (5.0) | 900 (4.7) | 900 (4.4) | 880 (8.5) |  | -80 (8.4) | -80 (8.6) | -79 (8.1) | -79 (7.9) | -86 (5.1) |
|  |  |  |  |  |  |  |  |  |  |  |  |  |
|  |  | **Background Material** | | | | |  | **Polystyrene** | | | | |
|  |  |  |  |  |  |  |  |  |  |  |  |  |
| 24 |  | 103 (1.0) | 103 (0.9) | 103 (1.0) | 103 (1.0) | 101 (1.0) |  | -27 (3.3) | -27 (3.3) | -27 (3.1) | -27 (3.0) | -30 (2.3) |
| 19 |  | 101 (0.8) | 101 (0.8) | 101 (0.9) | 101 (0.8) | 99 (0.6) |  | -28 (4.0) | -28 (3.8) | -28 (4.0) | -28 (3.9) | -29 (2.8) |
| 12 |  | 102 (1.0) | 103 (0.8) | 102 (1.0) | 102 (0.9) | 100 (0.9) |  | -28 (1.9) | -27 (1.7) | -28 (1.6) | -28 (1.5) | -30 (1.4) |
| 6 |  | 101 (1.2) | 101 (1.1) | 101 (1.1) | 101 (1.1) | 99 (0.9) |  | -31 (3.3) | -30 (3.0) | -31 (3.1) | -31 (3.0) | -33 (2.7) |
| 3 |  | 101 (1.5) | 101 (1.2) | 101 (1.4) | 101 (1.4) | 98 (1.0) |  | -27 (4.4) | -26 (3.5) | -27 (4.3) | -27 (4.2) | -29 (4.5) |
| 2 |  | 103 (1.6) | 103 (1.6) | 103 (1.6) | 103 (1.6) | 98 (1.7) |  | -23 (2.5) | -23 (2.4) | -23 (2.2) | -23 (2.1) | -28 (3.0) |
| 1 |  | 102 (1.8) | 102 (1.7) | 102 (1.7) | 102 (1.6) | 95 (1.6) |  | -25 (7.7) | -25 (7.7) | -25 (7.1) | -25 (6.7) | -29 (5.6) |
|  |  |  |  |  |  |  |  |  |  |  |  |  |
